# Carbofuran occupational dermal toxicity, exposure and risk assessment†

**DOI:** 10.1002/ps.2270

**Published:** 2011-08-10

**Authors:** Derek W Gammon, Zhiwei Liu, John M Becker

**Affiliations:** 1 FMC CorporationEwing, NJ, USA

**Keywords:** carbofuran, carbamate, occupational, risk assessment, dermal absorption, rat toxicity

## Abstract

**BACKGROUND:**

Carbofuran is a carbamate insecticide that inhibits AChE. Although toxic by ingestion in mammals, it has low dermal toxicity, with relatively few confirmed worker illnesses. This risk assessment describes its time of onset, time to peak effect and time to recovery in rats using brain AChE inhibition in acute and 21 day dermal studies; *in vitro* rat/human relative dermal absorption for granular (5G) and liquid (4F) formulations; occupational exposure estimates using the Pesticide Handlers' Exposure Database and Agricultural Handlers' Exposure Database (PHED/AHED).

**RESULTS:**

The point of departure for acute risk calculation (BMDL_10_) was 6.7 mg kg^−1^ day^−1^ for brain AChE inhibition after 6 h exposure. In a 21 day study, the BMDL_10_ was 6.8 mg kg^−1^ day^−1^, indicating reversibility. At 75 mg kg^−1^ day^−1^, time of onset was ≤30 min and time to peak effect was 6–12 h. Rat skin had ca tenfold greater dermal absorption of carbofuran (Furadan® 5G or 4F) than human skin. Exposure estimates for 5G in rice and 4F in ten crops had adequate margins of exposure (>100).

**CONCLUSION:**

Rat dermal carbofuran toxicity was assessed in terms of dose and time-related inhibition of AChE. Comparative dermal absorption in rats was greater than in humans. Worker exposure estimates indicated acceptable risk for granular and liquid formulations of carbofuran. Copyright © 2011 Society of Chemical Industry

## INTRODUCTION

Carbofuran is an *N*-methyl-carbamate insecticide/nematicide that has been used worldwide for about 40 years. It is systemic in plants and has a wide variety of crop uses, being effective in the control of both soil-dwelling and leaf-eating insects. Like the organophosphate insecticides, carbamates are inhibitors of acetylcholinesterase (AChE) and have high acute oral toxicity to mammals. However, unlike organophosphates, carbamates reversibly inhibit AChE^1^–^3^ and have considerably lower dermal toxicity,^1^ giving them a lower propensity to cause worker exposure illnesses, where the principal route of exposure is dermal. Occasional cases of worker illness have typically resulted from lack of adherence to label directions.^4^

Carbofuran technical is classified by the World Health Organization as highly hazardous, category 1b (rat oral LD_50_ 5–50 mg kg^−1^). However, this classification belies the fact that the technical product has a better safety profile by the dermal route (LD_50_ of > 1000 mg kg^−1^ in rat and > 2000 mg kg^−1^ in rabbit^1^) and would be classified as category II or III under WHO guidelines (moderately or slightly hazardous). As much as such classifications are helpful in standardizing comparisons of technical product, they are less relevant to actual agricultural settings. Growers are exposed to formulations and not technical product, and therefore the toxicity of the end-use formulation tends to be more relevant as a basis for establishing appropriate warnings to users. Granular carbofuran, containing 3 or 5% of the active ingredient, has much less toxicity than carbofuran technical via the dermal or oral routes. Toxicity by the inhalation route is largely dependent on the friability or dustiness of the granule, dusty granules having a greater likelihood of manifesting toxicity by inhalation. Thus, it is important for manufacturers and regulatory agencies to set a standard for low dust in the interest of worker safety and for responsible manufacturers to adhere to these standards.

Worker exposure risk assessments typically involve the calculation of a margin of exposure (MOE) by dividing a toxicological point of departure (POD), such as a no-observed-effect level (NOEL) or benchmark dose (BMD), by an exposure estimate. An MOE above 100 is generally considered to be adequate to protect a person from the toxic effects of a pesticide whenever the POD is based on an animal experiment. Key aspects such as time of onset, time to peak effect and time to recovery contribute to an understanding of the toxicodynamics of a carbamate insecticide. Also, a comparative rat/human dermal penetration study can contribute to a more accurate extrapolation from the test animal to humans.

This paper describes the results of studies on the dermal toxicity of carbofuran and the relevance of those results to worker risk for both granular and liquid formulations. A 21 day dermal study and acute studies of time to peak effect, onset of effect and reversibility, as well as skin absorption, are reported for two formulations (Furadan® 5G and Furadan® 4F). Occupational exposure was estimated using the Pesticide Handlers' Exposure Database (PHED)^5^ and the Agricultural Handlers' Exposure Database (AHED),^6^ standard databases in the United States. The results were then subjected to a worker risk analysis, the results of which shed light on the safety of carbofuran use as a liquid and granular formulation.

## MATERIALS AND METHODS

During 2009, three acute studies and one 21 day study were conducted at WIL Research Laboratories, Ashland, Ohio,^7^–^10^ in an attempt better to define the toxicology of carbofuran in the rat after dermal exposure. Groups of rats (10 sex^−1^ time interval^−1^) were exposed for specific times to carbofuran technical, applied as an aqueous paste, followed by washing of the treatment site for the removal of carbofuran residue. Initially, a dose response was developed for brain and red blood cell (RBC) AChE inhibition following a 6 h dermal exposure.^7^ Because 6 h was within the time period for maximum brain AChE inhibition, the above study was used to determine a BMDL_10_ for brain AChE inhibition that was considered to be suitable for risk assessment. A dose was selected (75 mg kg^−1^ day^−1^) that consistently gave ≥ 20% inhibition of RBC and brain AChE in both sexes for two follow-up studies that examined the time course of inhibition. In the time-of-onset study, groups of rats (10 sex^−1^ time^−1^) were exposed to 75 mg kg^−1^ day^−1^ for 0.5, 1.5, 3.0 and 6.0 h, followed by skin washing for removal of carbofuran residue, with additional groups assayed at 24 h after the 6.0 h exposure.^8^

Time to peak effect and time to recovery were studied using groups of rats dosed dermally at 75 mg kg^−1^ day^−1^ for 6 h, followed by skin washing.^9^ Then, AChE activity was measured at 6, 7.5, 9, 12, 24, 48 and 72 h after initial exposure. Recovery of AChE activity after acute exposure was slower than expected, so a 21 day study was conducted, with double washing of each rat at the end of each day's exposure period (6 h).^10^

In order to better translate to humans, relative dermal absorption of carbofuran was assessed using carbofuran with rat and human skin tissue *in vitro.* Finally, potential worker exposure was estimated, using PHED^5^ and AHED^6^ computer models and subsequent risk-assessment-derived MOEs for typical uses of carbofuran, following dermal exposure.

All studies in this series were conducted according to the current USEPA FIFRA GLP standards.

### Assessment of the dermal toxicity of carbofuran technical in the rat^7–10^

At WIL Research Laboratories LLC, Ashland, Ohio, carbofuran technical (98.4% purity) was administered to the shaved skin of adult rats of both sexes as a paste, mixed with deionized water just prior to application. Based on the body weight of each rat, the appropriate amount of carbofuran was weighed into a weigh-boat so that each rat would get the specified dosage in mg kg^−1^ day^−1^. In the first acute dermal study, rats were exposed to carbofuran for 6 h at 0, 25, 75, 150 and 250 mg kg^−1^. The time-course studies were conducted at 75 mg kg^−1^ day^−1^, the LOEL in both sexes from the initial acute study. In a 21 day dermal study, rats were dosed at 0, 20, 50 and 100 mg kg^−1^ day^−1^. Groups of rats (10 sex^−1^ dose^−1^) were administered an aqueous paste of carbofuran to the clipped dorsum for 6 h, in the same way as for the acute dermal studies, and the process was repeated daily on days 0 to 20.

Animals used were ca eight-week-old Crl:CD(SD) rats from Charles River Laboratories, Inc., Raleigh, North Carolina. The rats were acclimated for 2 weeks at WIL Laboratories, Inc. Body weights at the start of dosing were 219–310 g (males) and 164–230 g (females). Areas of skin were clipped from the back of each rat on the day prior to dosing, such that the area dosed with carbofuran was ca 10% of the body surface area. Control groups were administered ca 3–4 mL of vehicle (water). After the dosing of each rat, the application site was covered with a porous gauze wrap around the trunk, secured with elastic Co-Flex® bandage and held in place with non-irritating tape. Elizabethan collars were placed on all rats following dosing to prevent oral ingestion. At the end of each treatment period (usually 6 h), dressings and collars were removed, and the test sites were gently washed (distilled water, 1% baby shampoo, distilled water) using moist disposable paper towels, followed by drying with dry towels. In order to remove the carbofuran residue from the skin surface more completely, the washing process for the rat skin was repeated twice (compared with the acute studies) after each daily 6 h exposure for the 21 day study. Collars were replaced on animals except for those that were euthanized (carbon dioxide) for AChE determination at scheduled time points.

At scheduled time points (see Section 2), rats were dissected for collection of blood (ca 1 mL, vena cava), in heparinized test tubes, and brain, in 1% Triton® X-100. All samples were maintained on ice until further processing to ensure minimal reactivation of AChE. Measurement of cholinesterase activity started within 1 h of sample collection, a period that was found to avoid significant reactivation of AChE in a previous validation experiment using another carbamate, propoxur.^7^–^10^ RBC and whole-brain AChE activity were measured by a modified Ellman reaction,^11^, ^12^ using acetylthiocholine as a substrate.

The BMDL_10_ for AChE inhibition was calculated using USEPA's benchmark dose software (BMDS 2.1.2), in place of a NOEL. The Hill model consistently gave the best fit of data and was used for the BMD calculations.

#### Dermal absorption study of carbofuran in Furadan® 5G (5%) using human and rat skin *in vitro*^13^

A dermal absorption/penetration study was conducted by ADME, Vergese, France.^13^ Dermatomed human skin samples (mean thickness 247 ± 80 µm) were obtained from the abdomen of two female Caucasians, aged 31 and 48, and stored at − 20 °C. Rat skin samples (mean thickness 379 ± 15.5 µm) were obtained from two male hairless rats, aged 7 weeks, and stored at − 20 °C. Two skin samples were tested from each subject. Skin samples were mounted in a chamber bathed in static receptor fluid containing NaCl (9 g L^−1^) and PBS buffer (9.6 g L^−1^), plus water containing 5% acetone, at pH 6.5–7.0. A flowing water jacket maintained the temperature at 32 ± 1 °C. Skin integrity was assessed by measuring water loss from the *stratum corneum*, and this was found to be within acceptable limits. Carbofuran was quantified using a validated GC/MS method. Dosing used crushed 5G granules containing 6.2% carbofuran (measured) at a level of ca 10 mg cm^−2^, without dilution.

The skin chamber was humidified with water before dosing so as to mimic the presence of sweat. The receptor fluid was sampled on six occasions within the 24 h study duration, and carbofuran was measured. The granules were left on the skin for 8 h before washing with humidified cotton, water and Tween® 80. The amount of carbofuran was measured at 24 h in samples of receptor fluid, epidermis plus dermis, stratum corneum and skin excess, and recovery was recorded.

#### Dermal absorption study using human and rat skin *in vitro* with [^14^C]-carbofuran in Furadan® 4F^14^ (4 lb US gallon^−1^)

Another carbofuran dermal absorption/penetration study was conducted by Stratacor Inc., Richmond, California.^14^ Freshly excised human skin samples (*n* = 18) were obtained from the Cooperative Human Tissue Network (NIH, CHTN, Vanderbilt University Medical Center, Nashville, Tennessee) and dermatomed to 250–500 µm thickness (six samples from each of three donors). Similar samples of rat skin were also obtained (250–350 µm). An area of ca 0.8 cm^2^ of skin was exposed to treatment. Each skin sample was mounted into a chamber with the visceral side bathed in flowing receptor fluid containing tissue culture medium (RPMI formula 1640) plus NaHCO_3_, Hepes buffer, 4% BSA and gentamicin (50 mg L^−1^). Prior to each experiment, the integrity of each skin preparation was tested by applying [^3^H]-water; if the receptor fluid contained more than 3000 dpm after 1 h of incubation, the skin sample was assumed to be damaged and was discarded. [^14^C]-Carbofuran (Furadan® 4F) was phenyl UL at a specific activity of 55.4 mCi mmol^−1^, with a purity of 96.9%, and [^14^C]-piperonyl butoxide, also phenyl UL labeled, had a specific activity of 36.5 mCi mmol^−1^ and a purity of 96.5%. Dosing was conducted to achieve a chemical dose of ca 5900 µg cm^−2^ of carbofuran (both concentrated and dilute solutions) or 100 µg cm^−2^ of piperonyl butoxide, with a [^14^C] dose of ca 2 220 000 DPM cm^−2^.

[^14^C]-Carbofuran or positive control ([^14^C]-piperonyl butoxide) was applied at ca 10 mg 0.8 cm^−2^, and samples of receptor fluid were taken at intervals over 24 h. After 8 h, the clamp and donor chamber were removed from the skin surface, and excess material was removed from the skin surface using soap, water and dry wipes. Following decontamination, the donor chamber and clamp were repositioned on the skin surface. At 24 h after application, the clamp and donor chamber were again removed from the skin surface, and human skin was then tape stripped (twice) and the epidermis and dermis were separated. For rat skin, tape strips were not used, as this process had been found to damage rat skin. For human and rat skin, a conservative estimate of skin absorption was obtained by adding the total [^14^C] in the skin (epidermis, dermis and tape strips for human skin) to that found in the receptor fluid. Because of likely loss of epidermis residues through washing *in vivo*, a less conservative calculation combined the [^14^C] residues found in dermis with that in receptor fluid.

### Occupational exposure estimation scenarios

#### Occupational exposure for granules to rice

The estimation of occupational exposure used PHED for Furadan® 3G. The major application scenarios for workers applying the product were as follows:

1. mixing/loading and application of granular: ‘push-type’ granular spreader (open pour) on rice;
2. mixing/loading of granular: open mixing/loading for solid broadcast spreader application on rice;
3. application of granular: solid broadcast spreader, open cab, on rice.

The occupational exposure assessments were based on baseline personal protective equipment (PPE) as follows:

1. mixing/loading of granular: an open mixing and loading system needs minimum PPE—a single layer, no gloves or respirator;
2. solid broadcast spreader: an open cab with a single layer, no gloves or respirators.

#### Occupational exposure for liquid to vegetables and row crops

The estimation of occupational exposure (mixer/loader/applicator: M/L/A) used AHED and PHED for Furadan® 4F. The major application scenarios for workers applying the liquid product were as follows:

1. mixing/loading and application of liquids: aerial/groundboom canola, mustard, corn;
2. mixing/loading and application of liquids: groundboom sunflower, green pepper, potato, sugar beet, strawberry, raspberry, rutabaga, turnip.

The occupational exposure assessments were based on different levels of personal protective equipment (PPE) as follows:

1. mixing/loading of liquids: an open mixing and loading system needs maximum PPE—chemical-resistant coveralls over a single layer, chemical-resistant gloves and respirator;
2. groundboom application: a closed cab with chemical-resistant coveralls over a single layer, no gloves or respirators;
3. aerial application: a single layer (long-sleeved shirt and long trousers), with no gloves or respirators.

### Calculations for daily amount exposed

The values of ‘daily area treated’ for each application method, based on USEPA's Health Effects Division's Science Advisory Council for Exposure, policy number 9 of ‘Standard values for daily acres treated in agriculture’, were the same as those used in Canada by the Pest Management Regulatory Agency (PMRA).

### Calculations for occupational exposure

The PHED^5^ scenario used for Furadan® 3G was ‘open mixing/loading of granular’, ‘open cab solid broadcast spreader application’, ‘push-type granular spreader mixing/loading and application’. For Furadan® 4F, ‘enclosed cab aerial application of liquid formulations’ and two AHED^6^ scenarios of ‘open pour mixing and loading of liquid formulations’ and ‘open cab groundboom application of liquid sprays’ were used. In the scenario of ‘closed cab groundboom application of liquids’, because AHED™ does not have this specific scenario, a protection factor of 90% was applied for extrapolation from ‘open cab groundboom application’ to ‘closed cab groundboom application’. The 90% protection factor is considered very conservative (health protective) because it results in a fivefold higher exposure estimate (10% versus 2%) than the protection factor of 98% as recommended for the extrapolation from ‘open cabs’ to ‘enclosed cabs’.^6^

### Worker risk assessment

The MOEs for the dermal route of exposure were calculated using the following equation:


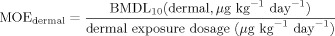


## RESULTS

### Dose selection for acute effects

**Figure 1:**
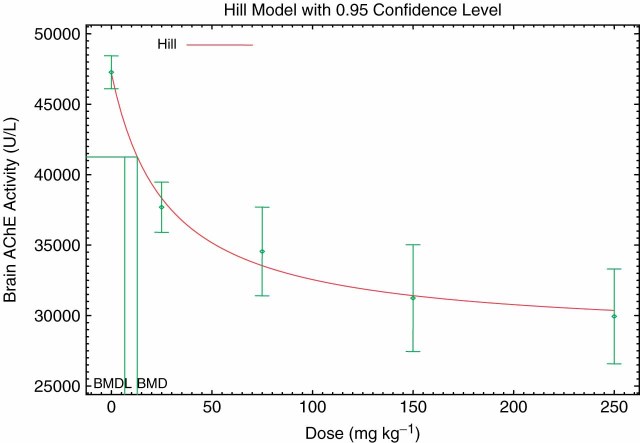
Inhibition of brain AChE activities following acute dermal exposure to carbofuran technical for 6 h.^2^.

Groups of ten rats of each sex were exposed to carbofuran dermally for 6 h at dosages of 0, 25, 75, 150 and 250 mg kg^−1^ day^−1^,^7^ and AChE activity was measured for RBC and brain (Table 1, Fig. 1). Both sexes showed dose-dependent inhibition of AChE, with the exception of RBC AChE inhibition in females, with few clinical signs. A BMDL_10_ of 6.7 mg kg^−1^ day^−1^ was obtained for brain AChE inhibition from this study (Fig. 1). Brain AChE was inhibited by a greater percentage than RBC AChE at three of four dosages in males and four of four dose levels in females. Therefore, brain AChE inhibition was considered to be a more reliable indicator of toxicity than RBC AChE inhibition.

**Table 1:** AChE activity (% inhibition) in the rat after dermal exposure to carbofuran (6 h)^a^

Dose (mg kg^−1^ day^−1^)	Male RBC	Male brain	Female RBC	Female brain	Combined sexes RBC	Combine sexes brain
0	3303 ± 817	47 768 ± 2227	3148 ± 553	46 768 ± 2756	3226 ± 683	47 268 ± 2492
25	2389 ± 454*	37 826 ± 4444**	2599 ± 646	37 553 ± 3278**	2494 ± 554 **	37 689 ± 3803**
	(27.7%)	(20.8%)	(17.5%)	(19.7%)	(22.7%)	(20.3%)
75	2410 ± 912*	32 391 ± 7908**	2517 ± 818	36 702 ± 4761**	2464 ± 845**	34 547 ± 6727**
	(27.0%)	(32.2%)	(20.0%)	(21.5%)	(23.6%)	(26.9%)
150	2133 ± 959**	30 199 ± 9543**	2630 ± 724	32 271 ± 6733**	2382 ± 866**	31 235 ± 8108**
	(35.4%)	(36.8%)	(16.5%)	(31.0%)	(26.2%)	(33.9%)
250	2059 ± 516**	28 523 ± 6685**	2379 ± 762	31 354 ± 7738**	2219 ± 654**	29 939 ± 7186**
	(37.7%)	(40.3%)	(24.4%)	(33.0%)	(31.2%)	(36.7%)

* *P* < 0.05 different from control.

** *P* < 0.01 different from control.

a Mean enzyme activity U/L ± standard deviation (percentage inhibition compared with control).^2^

From the dose response it was determined that 75 mg kg^−1^ day^−1^ was suitable for studying the time course of AChE inhibition because it was the lowest dose that caused ≥ 20% inhibition of brain and RBC AChE in both sexes at 6 h. This low dosage would allow a better assessment of recovery than a higher one. Inhibition of rat brain AChE of 20% has recently been used by WHO/FAO to establish an acute RfD for dietary risk assessment of carbofuran.^2^

### Time to onset and time to peak effect

In general, the inhibition of both brain and RBC AChE increased with duration of dermal exposure at 75 mg kg^−1^ day^−1^.^8^ The inhibition of brain AChE was greater than RBC AChE inhibition at all five time points, for both males and females. Male and female rats showed similar degrees of enzyme inhibition (Table 2). Because AChE was inhibited by 22–33% after 30 min of exposure, the time of onset for AChE inhibition was 30 min or less. Similarly, AChE inhibition after 6 h exposure was greater than after 3 h, so the time to peak effect was probably 6 h or greater.

**Table 2:** AChE activity (% inhibition) in the rat after dermal exposure (0–6 h) with carbofuran at 75 mg kg^−1^^a^

Exposure duration (h)	Male RBC	Male brain	Female RBC	Female brain	Combined sexes RBC	Combined sexes brain
0	4314 ± 883	49 148 ± 1698	4285 ± 748	48 930 ± 2215	4300 ± 797	49 039 ± 1924
0.5	3253 ± 656*	33 983 ± 5890*	3304 ± 752*	32 731 ± 6569*	3279 ± 687**	33 357 ± 6106**
	(24.6%)	(30.9%)	(22.9%)	(33.1%)	(23.7%)	(32.0%)
1.5	2811 ± 718*	29 817 ± 5613*	3035 ± 692*	31 178 ± 7357*	2923 ± 696**	30 498 ± 6407**
	(34.8%)	(39.3%)	(29.2%)	(36.3%)	(32.0%)	(37.8%)
3.0	2948 ± 797*	32 236 ± 6557*	2808 ± 603*	31 149 ± 6289*	2878 ± 692**	31 693 ± 6278**
	(31.7%)	(34.4%)	(34.5%)	(36.3%)	(33.1%)	(35.4%)
6.0	2418 ± 771*	26 094 ± 8434*	2579 ± 1120*	28 846 ± 5513*	2499 ± 940**	27 470 ± 7077**
	(44.0%)	(46.9%)	(39.8%)	(41.0%)	(41.9%)	(44.0%)
6.0 + 18 h recovery	3134 ± 973*	30 172 ± 5369*	2893 ± 861*	32 047 ± 6191*	3014 ± 898**	31 110 ± 5721**
	(27.4%)	(38.6%)	(32.5%)	(34.5%)	(30.1%)	(36.6%)

* *P* < 0.05 different from control.

** *P* < 0.01 different from control.

a Mean enzyme activity U/L ± standard deviation (percentage inhibition compared with control).^3^

After 6 h of dermal exposure, followed by skin washing and 18 h for potential recovery, partial recovery was observed. The inhibition of brain AChE had fallen from 41–47% to 35–39%. For RBC AChE, which was more variable, similar time of onset and time to peak effect were observed, along with a similar partial recovery at 24 h (inhibition 27–33% versus 40–44% at 6 h).

### Time to peak effect and time to recovery

**Figure 2:**
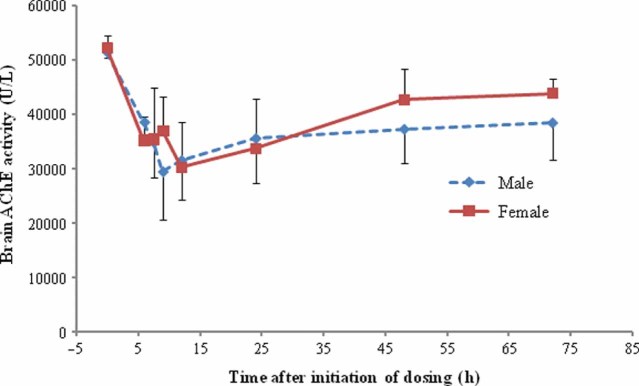
Effect of dermal carbofuran on rat brain AChE activity, U/L (mean ± SD).^4^.

As in previous studies, there was a substantial inhibition (25–32%) of brain AChE at 6 h.^9^ Because of variability, a statistically significant inhibition (*P* < 0.01) of brain AChE in males and females was observed only at 12 h. It is likely that the time to peak effect occurred during the 6–12 h plateau, as there was no significant difference in the brain AChE inhibition recorded at 6, 7.5, 9 and 12 h (Fig. 2). After skin washing at 6 h, a similar degree of brain AChE inhibition remained at 7.5, 9, 12 and 24 h. Partial recovery had occurred after 48 and 72 h.

**Figure 3:**
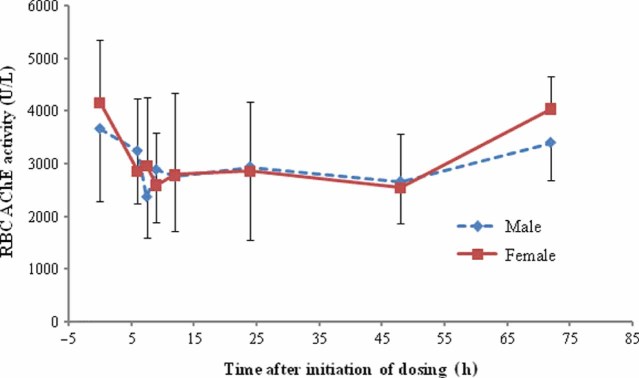
Effect of dermal carbofuran on rat RBC AChE activity, U/L (mean ± SD).^4^.

The inhibition of RBC AChE was also pronounced at 6 h (11–31%) and formed a plateau until 48 h following dermal exposure. By 72 h, recovery of enzyme activity was essentially complete, as inhibition was only 7.3% in males or 2.8% in females (Fig. 3). The inhibition of brain AChE was greater and longer lasting than RBC AChE inhibition at six of seven time points in males and at five of seven time points in females.

Thus, following dermal dosing of the rat for 6 h, inhibition of AChE (brain and RBC) had peaked by 6 h and remained depressed to a similar degree until 12 h and possibly beyond. Because of the plateau of inhibition that occurred following 6 h of dermal exposure, it is reasonable to consider exposure for 6 h to be the time to peak effect for conducting reversibility studies. No significant increase in AChE inhibition took place after 6 h, but recovery from inhibition appeared to be slow. The method of washing the rat skin was considered to be a confounding factor for the apparently slow recovery.

### Twenty-one day dermal dose response

Because of the limited reversibility of brain and RBC AChE inhibition in the acute dermal studies, it was not clear whether the toxic effects of carbofuran following dermal dosing would reverse within 24 h of the initiation of dosing. Therefore, to assess possible risks following repeated daily worker exposure, a 21 day repeated dose dermal study was conducted.^10^ Double washing of the rat skin (compared with the acute studies) was performed at the end of each daily 6 h exposure for more complete removal of the carbofuran residue from the skin surface.

**Figure 4:**
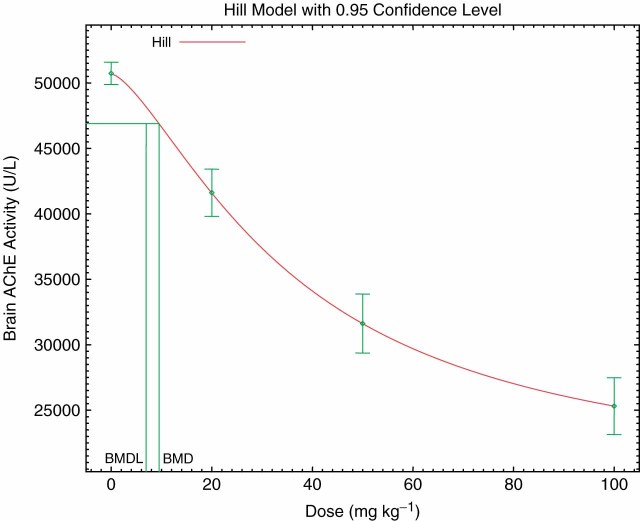
Inhibition of brain AChE activities after dermal exposure to carbofuran technical for 21 days (6 h day^−1^).^5^.

During the 21 day study there were no notable carbofuran-related clinical signs. Following the final 6 h exposure, brain and RBC AChE activities were measured. AChE inhibition was significant (*P* < 0.01) in all dose groups in a dose-dependent manner, but the LOEL for 20% inhibition was between 20 and 50 mg kg^−1^ day^−1^ (Table 3). Slightly greater inhibition of RBC AChE compared with brain AChE was observed. A BMDL_10_ of 6.8 mg kg^−1^ day^−1^ (BMD_10_ = 9.1 mg kg^−1^ day^−1^) was obtained for brain AChE inhibition from this study (Fig. 4). This study demonstrated that full recovery occurred between daily doses, as there was no significant difference in toxicity between the acute studies and the 21 day study. It is probable that this resulted from more thorough decontamination in the 21 day study.

**Table 3:** AChE activity (% inhibition) in the rat after dermal exposure to carbofuran (21 days)^a^

Dose (mg kg^−1^ day^−1^)	Male RBC	Male brain	Female RBC	Female brain	Combined sexes RBC	Combined sexes brain
0	2977 ± 665	50 957 ± 2010	2758 ± 780	50 495 ± 1686	2868 ± 714	50 726 ± 1821
20	2045 ± 352**	42 666 ± 4179**	2351 ± 1145	40 546 ± 3179**	2198 ± 857**	41 606 ± 3742**
	(31.3%)	(16.3%)	(14.8%)	(19.7%)	(23.1%)	(18.1%)
50	1561 ± 352**	33 580 ± 4867**	1216 ± 324**	29 652 ± 3662**	1389 ± 374**	31 616 ± 4676**
	(47.6%)	(34.1%)	(55.9%)	(41.3%)	(51.6%)	(37.5%)
100	1242 ± 326**	26 266 ± 3089**	832 ± 330**	24 346 ± 5711**	1037 ± 382**	25 306 ± 4499**
	(58.3%)	(48.5%)	(69.8%)	(51.8%)	(63.5%)	(50.0%)

* *P* < 0.05 different from control.

** *P* < 0.01 different from control.

a Mean enzyme activity U/L ± standard deviation (percentage inhibition compared with control).^6^

### Dermal absorption of carbofuran

#### Measurement of dermal absorption of carbofuran in Furadan® 5G using human and rat skin *in vitro*

At a dose of 9.56 mg cm^−2^, the dermal absorption of carbofuran was 0.03 ± 0.02% for human skin over 24 h, including an 8 h exposure period (Table 4).^13^ This had all penetrated the skin into the receptor fluid, and very little was associated with the dermal residues. Using rat skin, the corresponding absorption figure, at a dose of 10.40 mg cm^−2^, was 0.31 ± 0.24%, of which 34% of the carbofuran was associated with the receptor fluid and 65% with the skin. The amount of carbofuran associated with the outer skin layer, the stratum corneum, was below the limit of quantification. Although a positive control was not performed in this study, the total recovery (97.83% for human and 92.43% for rat) was acceptable, according to OECD guidelines (number 428). It can be concluded that absorption of carbofuran from Furadan® 5G by rat skin was approximately tenfold greater than for human skin. The study was considered to provide complementary information to that in the EU review by Belgium.^15^

**Table 4:** Dermal absorption of carbofuran in human and rat skin exposed to Furadan® 5G

Species/dose level	Human/9.56 ± 0.48 mg cm^−2^	Rat/10.40 ± 0.34 mg cm^−2^
Skin excess (µg cm^−2^)	576.89 ± 35.54	590.73 ± 32.08
% applied dose	97.80 ± 1.38	92.07 ± 3.03
Stratum corneum (SC) (µg cm^−2^)	< LOQ	< LOQ
% applied dose	< LOQ	< LOQ
Epidermis + dermis (ED) (µg cm^−2^)	0.03 ± 0.03	1.30 ± 1.18
% applied dose	0.005 ± 0.005	0.20 ± 0.18
Receptor fluid (RF) (µg cm^−2^)	1.163 ± 0.117	0.682 ± 0.453
% applied dose	0.03 ± 0.02	0.106 ± 0.067
Dermal delivery (ED + RF) (µg cm^−2^)	0.193 ± 0.143	1.983 ± 1.616
% applied dose	0.03 ± 0.02	0.31 ± 0.24
Total recovery (% applied dose)	97.83 ± 1.40	92.43 ± 2.97

#### Measurement of dermal absorption of [^14^C]-carbofuran in Furadan® 4F using human and rat skin *in vitro*

**Figure 5:**
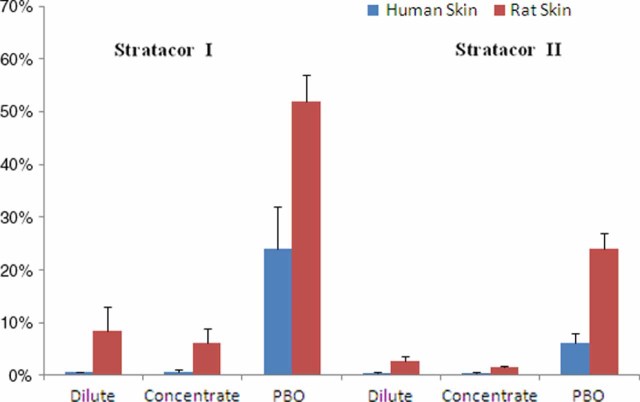
Percutaneous absorption of [^14^C]-carbofuran in Furadan® 4F and [^14^C]-piperonyl butoxide (PBO, 97 µg cm^−2^) applied to viable excised human versus rat skin (mean ± SD, *n* = 6). Stratacor I: dermal absorption (tape strips + epidermis + dermis + receptor fluid); Stratacor II: dermal penetration (dermis + receptor fluid).^10^.

At doses of 5400 and 6100 µg [^14^C]-carbofuran cm^−2^, values for dermal absorption through human skin were 0.64 ± 0.34% and 0.60 ± 0.11% respectively (*n* = 6 dose^−1^).^14^ For rat skin at 4700 and 6100 µg [^14^C]-carbofuran 0.8 cm^−2^, the corresponding dermal absorption values were 6.2 ± 2.6% and 8.4 ± 4.6%. As a positive control, [^14^C]-piperonyl butoxide absorption (at 100 µg cm^−2^) was also measured in both systems. Values of 24 ± 3% (human) and 52 ± 5% (rat), using *n* = 6 dose^−1^, were obtained. These human absorption values were above those reported in the literature, ensuring that carbofuran dermal absorption was not underestimated. All of the above calculations were based on radiolabel recovered in receptor fluid, dermis, epidermis and tape strips 24 h after dosing the skin. The results (Fig. 5) indicate that dermal absorption of carbofuran by rat skin is 9.7–14.0-fold greater than by human skin. Using a less conservative calculation of dermal absorption, i.e. combining receptor fluid and dermis, the difference between rat and human skin was less, 4.4–5.9-fold, with rat again absorbing more than human skin.

### Calculations of occupational exposure

The estimates of occupational exposure are summarized in Table 5 (3G) and Table 6 (4F). For 3G application, rice was the crop chosen for exposure assessment, whereas, for 4F, estimates include dermal exposure for several tasks on ten different crops. These are canola (rapeseed), sunflower, corn, mustard, green pepper, potato, sugar beet, raspberry, strawberry and turnip.

**Table 5:** Occupational exposure/risk (MOE) for tasks associated with Furadan® 3G applications to rice, using a single layer with no gloves^a^

Crop	Application scenario^b^	Application rate^c^ (g AI ha^−1^)	Area treated (ha day^−1^)	Dermal exposure^d^ (µg kg day^−1^)	MOE^e^
Rice	‘Push-type’ granular spreader (open pour)—M/L/A	1875	2	342.50	196
Rice	Solid broadcast spreader, open cab, Ag uses—open M/L	1875	80	39.68	1688
Rice	Solid broadcast spreader, open cab, Ag uses—A	1875	80	46.77	1433

a Mixer/loader: an open mixing and loading system. Applicator: a single layer (long-sleeved shirt and long trousers) with no gloves.

b M/L/A = Mixer/loader/applicator; M/L = mixer/loader; A = applicator.

c Maximum listed label application rate in g AI ha^−1^.

d Dermal exposure = (unit exposure × area treated × application rate) 70 kg^−1^.

e Based on a dermal BMDL_10_ of 67.0 mg kg^−1^ day^−1^ = 6.7 mg kg^−1^ day^−1^ × 10 (relative rat/human dermal penetration factor for Furadan® 5G and a target MOE = 100).

**Table 6:** Occupational exposure/risk (MOE) for tasks associated with Furadan® 4F applications to ten crops, using maximum personal protection equipment^a^

Crops	Application scenario^b^	Application rate^c^ (g AI ha^−1^)	Area treated (ha day^−1^)	Dermal exposure^d^ (µg kg^−1^ day^−1^)	MOE^e^
Canola (rapeseed)	Aerial—M/L	132	400	17.13	3795
Canola (rapeseed)	Aerial—A	132	400	7.29	8916
Canola (rapeseed)	Groundboom	132	100	0.16	411 459
Sunflower	Groundboom	132	100	0.16	411 459
Corn	Aerial—M/L	528	400	68.51	949
Corn	Aerial—A	528	400	29.15	2230
Corn	Groundboom	528	80	0.51	128 581
Mustard	Aerial—M/L	132	400	17.13	3795
Mustard	Aerial—A	132	400	7.29	8916
Mustard	Groundboom	132	100	0.16	411 459
Green pepper	Groundboom	528	30	0.19	342 883
Potato	Groundboom	528	80	0.51	128 581
Sugar beet	Groundboom	1123	30	0.40	161 213
Raspberry	Groundboom	1200	30	0.43	150 868
Strawberry	Groundboom	1200	30	0.43	150 868
Rutabaga, turnip	Groundboom	2520	60	1.81	35 921

a Mixer/loader. An open mixing and loading system with chemical-resistant coveralls over a single layer with chemical-resistant gloves and a suitable respirator. Groundboom applicator: a closed cab with chemical-resistant coveralls over a single layer (no gloves or respirators). Aerial applicator: a single layer (long-sleeved shirt and long trousers), no gloves or respirators.

b M/L = mixer/loader; A = applicator; groundboom—farmer groundboom application.

c Maximum listed label application rate in g AI ha^−1^.

d Dermal exposure = (unit exposure × area treated × rate) 70 kg^−1^.

e Based on a dermal BMDL_10_ of 65.0 mg kg^−1^ = 6.7 mg kg^−1^ day^−1^ × 9.7 (relative rat/human dermal penetration factor) and a target MOE of 100.

### Risk assessment for occupational exposure

The dermal toxicity endpoint (i.e. BMDL_10_ = 6.7 mg kg^−1^ day^−1^) used in this assessment was derived from an overall evaluation of three acute dermal toxicity studies.^7^–^9^ In a 21 day dermal toxicity study in rats, a BMDL_10_ of 6.8 mg kg^−1^ day^−1^ was determined.^10^ The dermal toxicity studies were considered to be appropriate for derivation of a toxicity endpoint for the short- and intermediate-term dermal exposures because the study was route specific. No route-specific absorption factor adjustment was required in this case because the risks from dermal exposure were assessed on the basis of a toxicity endpoint from a dermal toxicity study. However, the carbofuran *in vitro* comparative rat versus human dermal absorption studies showed that human skin has ca tenfold (5G) and 9.7–14.0-fold (4F) less dermal absorption of carbofuran in Furadan® compared with rat skin,^13^, ^14^ and these species differences have been incorporated into this assessment.

The short- and intermediate-term MOEs for mixing, loading and applying product in each typical exposure scenario were considered for Furadan® 3G and 4F label crop uses. The MOEs estimated from dermal exposure are summarized in Tables 5 and 6 (including a 9.7× relative rat versus human dermal absorption factor).

MOEs that are > 100 indicate a margin of safety greater than the level of concern, and those tasks are thus considered to be acceptable and not hazardous for workers. For 3G uses on rice, the calculated MOEs for mixing/loading and applying carbofuran were above 100, even without wearing gloves. For proposed uses of Furadan® 4F, 15 of 16 exposure scenarios had MOEs that were > 100, without adjusting for human/rat dermal absorption, so label uses of the product under these scenarios are considered to pose no unacceptable risks to workers while mixing, loading and applying the product. There was only one scenario (mixing/loading for aerial application to corn) that had an MOE of < 100, suggesting that further refinement was needed for this use scenario. The additional refinement of the 9.7-fold less relative dermal absorption in human versus rat skin results in the calculated MOE at 949 for the scenario of corn mixing/loading aerial. Therefore, all of the exposure scenarios are considered to be safe for workers when the human dermal absorption factor is used.

## DISCUSSION

After dermal administration of carbofuran technical to the rat, inhibition of brain and RBC AChE was recorded in the absence of clinical signs of toxicity. The time of onset was rapid, with significant AChE inhibition being observed within 30 min of the commencement of dosing. This onset time is similar to that of carbofuran following oral gavage dosing of the rat.^16^ At 75 mg kg^−1^ day^−1^, the peak effect appeared to occur between 6 and 12 h after dermal dosing, slightly longer than after oral gavage dosing.^16^ The relative inhibition of brain and RBC AChE was similar after dermal and oral dosing, but, in the case of acute dermal exposure, the brain enzyme was generally inhibited to a greater degree than the RBC AChE. After a 6 h exposure, the BMDL_10_ was 6.7 mg kg^−1^ day^−1^ for brain AChE, and this value was chosen for conducting risk assessments for dermal exposure. Recovery of AChE activity after a 6 h exposure was partial for brain and complete for RBC by 48–72 h. Because of residual inhibition of brain AChE at 24 h, a 21 day repeat dose study was conducted for better description of the effects of repeated exposure. In this study, which involved double washing after each day's exposure, slightly greater inhibition of RBC AChE was observed, compared with the brain enzyme, and a BMDL_10_ of 6.8 mg kg^−1^ day^−1^ was obtained for brain. It was concluded that the poor recovery of brain AChE in the acute experiments resulted from incomplete washing of the skin, such that dermal deposits acted as a reservoir of carbofuran during the recovery phases. This conclusion is supported by two earlier range-finding studies in which groups of rats were dosed dermally for up to 6 h at 250 or 500 mg kg^−1^ day^−1^,^17^ and at 750 mg kg^−1^ day^−1^,^18^ followed by measurement of brain and RBC AChE inhibition 3, 6, 6.5, 7, 8, 9 and 25 h after the start of dosing. The inhibition of AChE (brain and RBC) was between 50 and 63% for all (three) doses at all time points. The interpretation of these data was that, because dermal absorption usually declined (as a percentage of applied dose) as the dose increased, it is probable that the internal dose, as well as the AChE inhibition, was the same at the three dosages. In all of these studies, the only rats showing clinical signs of tremors, convulsions and salivation were individuals that had removed their collars and thus ingested carbofuran orally.

At the enzyme level, recovery of brain AChE from carbofuran inhibition is relatively rapid, as demonstrated by studies using gavage dosing. For example, at 0.5 mg kg^−1^, inhibition of rat brain AChE of ca 40% within 30–60 min of dosing had returned to normal by 24 h.^19^ Brain and RBC AChE were inhibited to similar degrees, with the latter inhibited slightly more than the former. In an oral gavage dose response study, carbofuran at 0.1–1.5 mg kg^−1^ inhibited AChE to similar degrees, along with causing corresponding depressions of motor activity.^20^ In a time-course study in immature rats, oral gavage dosing with carbofuran resulted in peak inhibition of AChE at 15–45 min (brain) and 15–180 min (RBC), with complete recovery of both enzymes by 24 h.^16^ Thus, although these studies demonstrated relatively rapid reversal of AChE inhibition by carbofuran compared with organophosphate inhibition, they all used oral gavage dosing, giving little indication of the reversibility of carbofuran effects after dermal exposure. The studies described in this paper suggest that recovery of AChE following dermal exposure also occurs within 24 h, especially with adequate washing after each exposure. Extrapolating to humans, this indicates that PPE and good hygiene are important to worker safety.

Dermal absorption of carbofuran in Furadan® 5G and 4F was measured, *in vitro*, using human and rat skin samples. The 5G is anticipated to be similar to the 3G, which was used in the occupational exposure calculations. The granular was absorbed less than the liquid formulation by both rat and human skin. Moreover, both formulations were absorbed more by rat than by human skin (ca tenfold). Dermal penetration of 4F included [^14^C] in receptor fluid plus dermis, whereas absorption additionally included [^14^C] retained in the epidermis. Penetration of carbofuran through rat skin was 4–6-fold greater than through human skin. The dermal absorption into skin was 9.7–14.0-fold greater in rat than in human skin. In addition, absorption of the positive control in these studies ([^14^C]-piperonyl butoxide) was greater than in literature reports, assuring that dermal absorption of [^14^C]-carbofuran was unlikely to be underestimated. EPA and other regulatory bodies have consistently taken into account the rat/human relative dermal penetration/absorption of a pesticide when conducting a risk assessment. For carbaryl,^21^ an *in vitro* dermal penetration factor of 2.8-fold was applied to the critical rat dermal BMDL_10_ following an *in vitro* comparative dermal study similar to the one described here for carbofuran.

The 3G granular formulation of carbofuran gave adequate MOEs (>100) for all tasks (M/L/A) in applications on rice, even without the most basic PPE, i.e. no gloves. Because rice is a crop on which carbofuran is used heavily, it is therefore anticipated that other crops will also have adequate MOEs. The use of the liquid 4F formulation, however, required maximum PPE for open cab and open system tasks, including a respirator, and gave lower MOEs than the use of 3G granular material, with minimal PPE. MOEs for 4F were nonetheless above 100 even without adjusting for relative rat/human dermal absorption in 15 of 16 scenarios. By including this factor, all of the scenarios had adequate MOEs.

## CONCLUSIONS

The dermal toxicity of carbofuran in relation to the risk of agricultural worker illness incidents was determined in this paper through a series of toxicity and dermal absorption studies. The rat was used to determine the time of onset, time to peak effect and time to recovery of brain AChE activity, using carbofuran technical. Using 75 mg kg^−1^ day^−1^ (giving ≥ 20% inhibition of both brain and RBC AChE), it was found that time of onset was within 30 min, time to peak effect was approximately 6 h and recovery of brain AChE activity was incomplete, even at 72 h. In a 21 day repeat-dose dermal study there was no evidence of increased toxicity, so recovery within 24 h was indicated. BMDL_10_ values of 6.7 and 6.8 mg kg^−1^ day^−1^ were obtained for brain AChE inhibition in acute (6 h) and 21 day studies respectively. Rat skin was found to be ca tenfold more permeable to carbofuran than human skin. Using PHED and AHED, granular carbofuran (Furadan® 3G) use on rice had MOE values above 100, even without wearing gloves, whereas, for Furadan® 4F, full PPE was required before the MOE was above 100. For this reason, liquid formulations of Furadan® are used only under controlled conditions.
